# Supplementary material for: Effective Diagnosis of Foot-And-Mouth Disease Virus (FMDV) Serotypes O and A Based on Optical and Electrochemical Dual-Modal Detection
Source: Biomolecules. 2021 Jun 5;11(6):841. doi: 10.3390/biom11060841 (PMC8229964; doi:10.3390/biom11060841)
Supplement: Supplementary file 1 [file biomolecules-11-00841-s001.zip › biomolecules-1205287-supplementary.pdf]

Supplementary information

Effective diagnosis of foot-and-mouth disease virus (FMDV) serotypes O and A based on optical and electrochemical du-al-modal detection

Yun-Jung Hwang[1,2], Kyung-Kwan Lee[1,3,4], Jong-Won Kim[1,4], Kwang-Hyo Chung[4], Sang-Jick Kim[5], Wan Soo Yun[2,*], Chang-Soo Lee[1,4,6,*]

[1] Bionanotechnology Research Center, Korea Research Institute of Bioscience and Biotechnology (KRIBB), Daejeon 34141, Korea; yunjung1758@gmail.com (Y.-J.H.); lkk@kribb.re.kr (K.-K.L.); kimjw@kribb.re.kr (J.-W.K.)
[2] Department of Chemistry, Sungkyunkwan University, Suwon 16419, Korea; wsyun87@skku.edu
[3] Department of Biomedical and Nanopharmaceutical Science, College of Pharmacy, Kyung Hee University, Seoul 02447, Korea
[4] Dignostics Platform Research Section, Electronics and Telecommunications Research Institute (ETRI), Daejeon 34129, Korea; hyo@etri.re.kr (K.-H.C.)
[5] Synthetic Biology and Bioengineering Research Center, Korea Research Institute of Bioscience and Biotechnology (KRIBB), Daejeon 34141, Korea; sjick@kribb.re.kr (S.-J.K.)
[6] Department of Biotechnology, University of Science & Technology (UST), Daejeon 34113, Korea
* Correspondence: cslee@kribb.re.kr (C.-S.L.); Tel.: +82-42-879-8446; wsyun87@skku.edu (W.S.Y.); Tel.: +82-31-299-4568

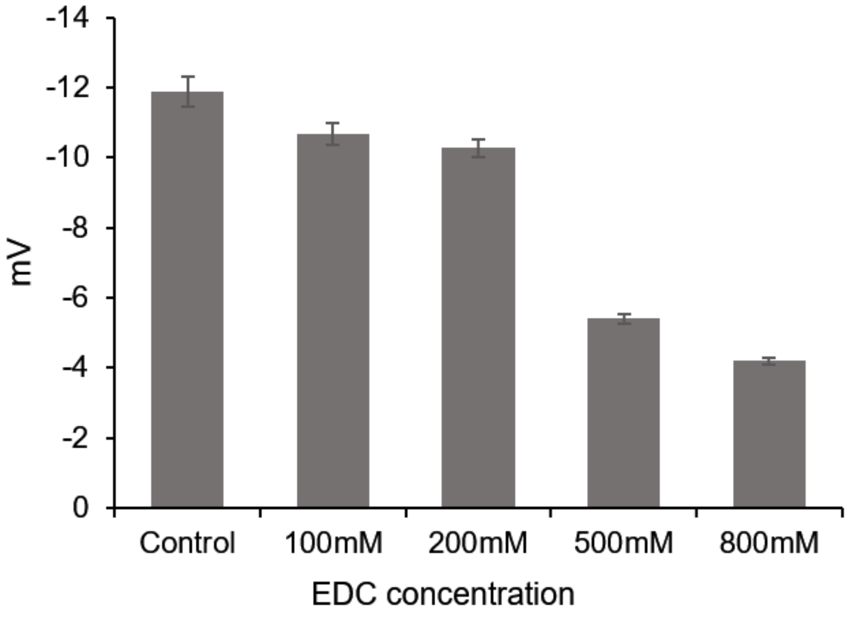

Figure S1. Effect of EDC activation assisted by NHS on the zeta potential of carboxylated MNPs.

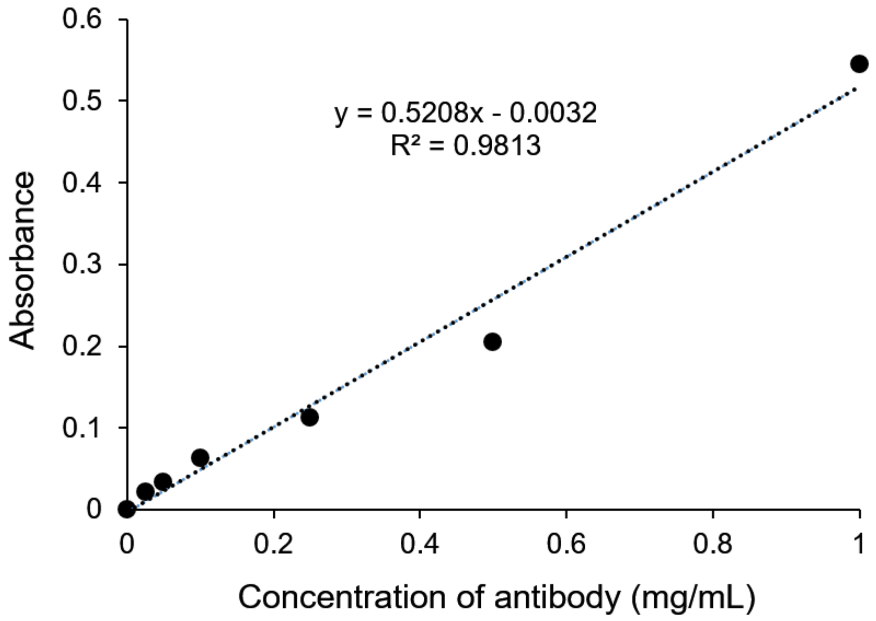

Figure S2. Calibration curve by the Bradford assay measured at 595 nm wavelength.

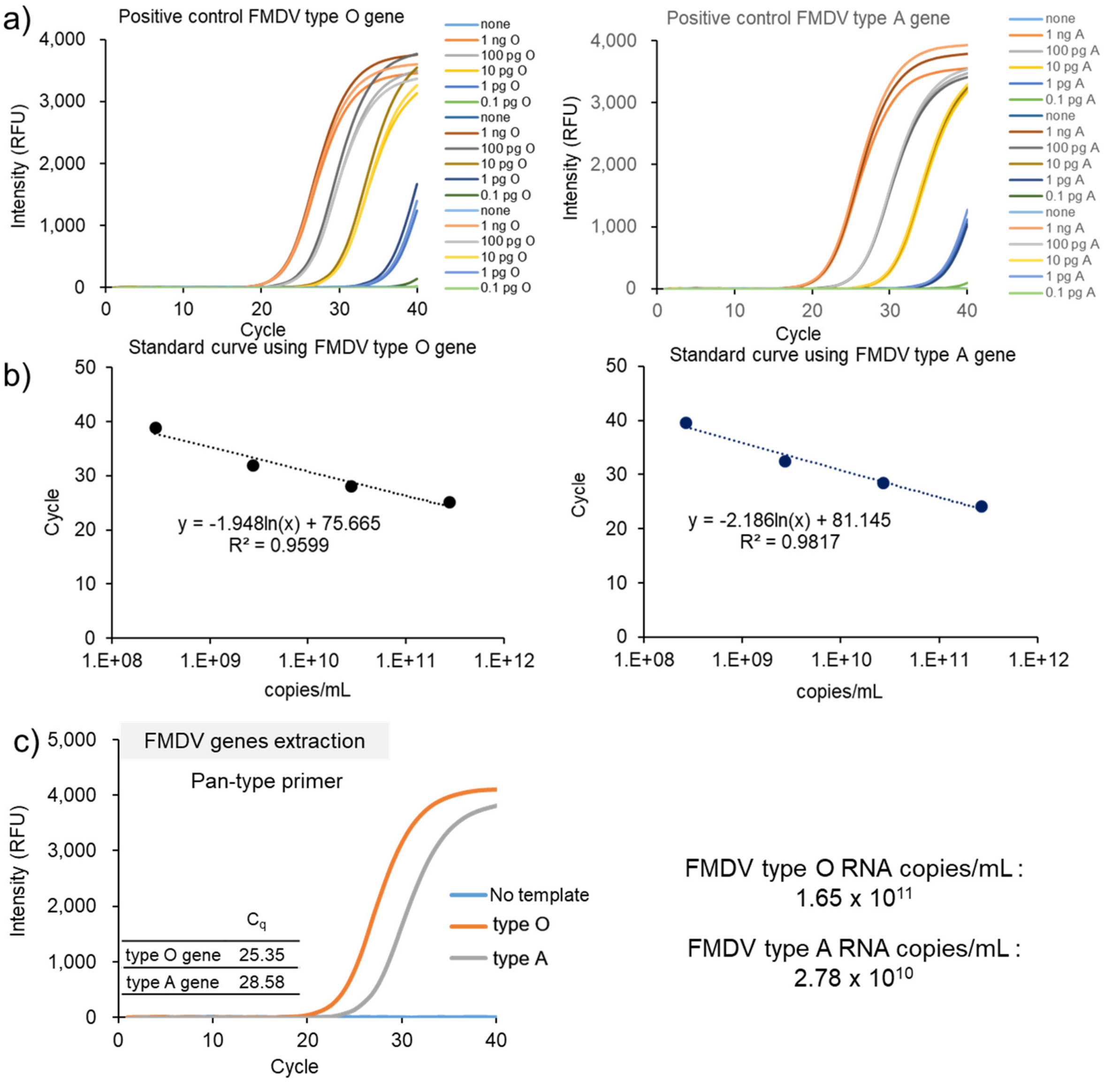

Figure S3. Calibration curve by Bradford assay measured at 595 nm wavelength. The process of obtaining FMDV concentration. (a) FMDV gene RT-qPCR results, (b) standard curve results according to FMDV gene RT-qPCR, (c) FMDV concentration results obtained by standard curve through extracted FMDV RNA.

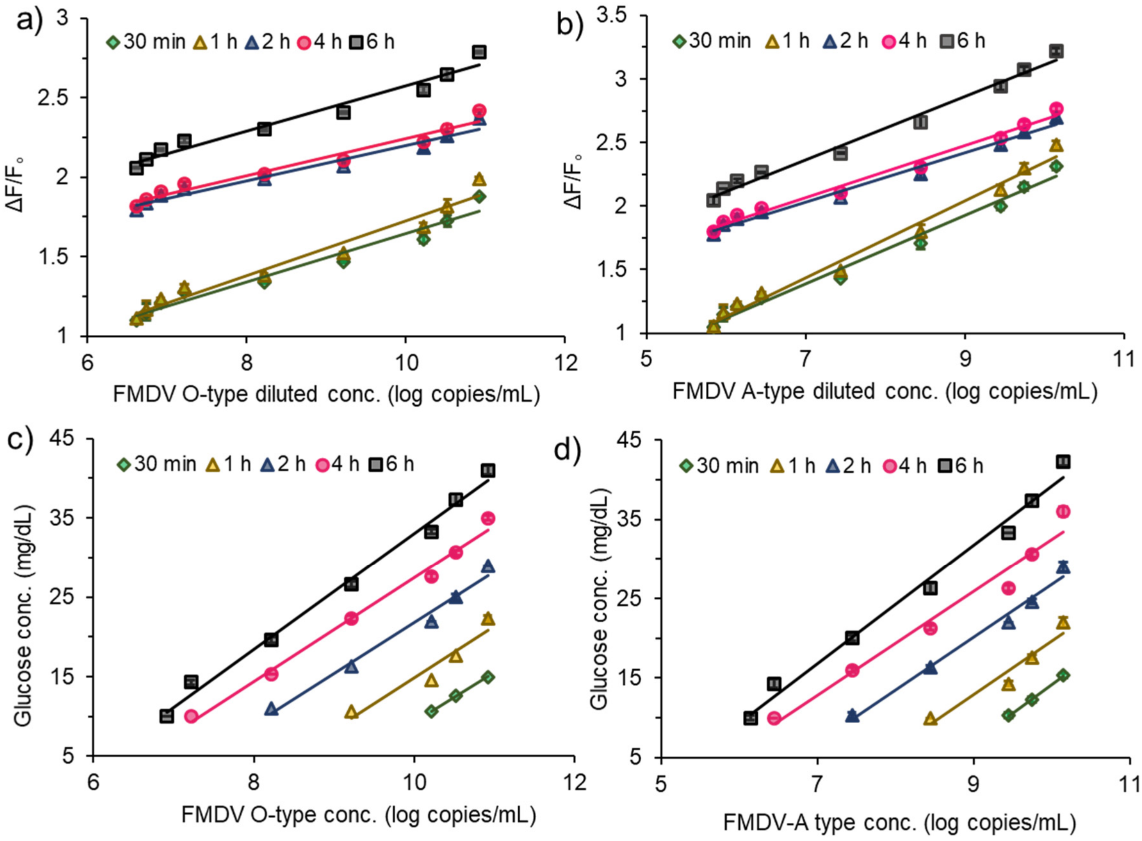

Figure S4. Sensitivity test by the time of the optical and electrochemical multimodal detection of FMDV serotype O and A. (a) The fluorescence measurement result of FMDV serotype O by time, (b) The fluorescence measurement result of FMDV serotype A by time, (c) The electrochemical measurement result of FMDV serotype O using glucometer by time, (d) The electrochemical measurement result of FMDV serotype A using glucometer by time.

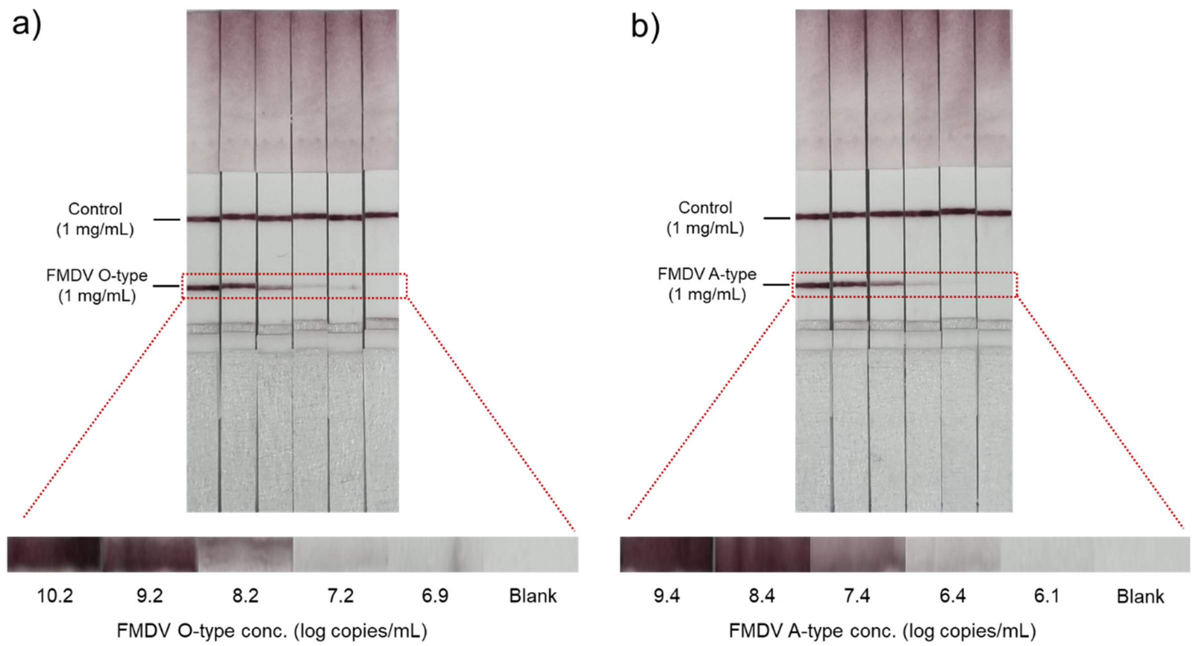

Figure S5. Sensitivity for FMDV O- and A-type using LFA kit. Results were quantified using Image J program. (a) LFA result for FMDV O-type, (b) LFA result for FMDV A-type.
